# Bend-resistant leaky multi-trench fiber with large mode area and single-mode operation

**DOI:** 10.1371/journal.pone.0203047

**Published:** 2018-08-30

**Authors:** Shaoshuo Ma, Tigang Ning, Li Pei, Jing Li, Jingjing Zheng, Xueqing He, Xiaodong Wen

**Affiliations:** 1 Key Lab of All Optical Network & Advanced Telecommunication Network of EMC, Institute of Lightwave Technology, Beijing Jiaotong University, Beijing, China; 2 College of Physics and Engineering, Qufu Normal University, Qufu, China; University of Cambridge, UNITED KINGDOM

## Abstract

A novel structure of modified multi-trench fiber (MTF) with characteristics of bend-resistance and large mode-area is proposed. In this structure, each low refractive-index trench of traditional MTF is broken by two gaps up and down. Numerical investigations show that the mode field area of 840 μm^2^ can be achieved with effective single-mode (SM) operation when the bending radius is 15 cm. Moreover, the high order mode (HOM) suppression of the proposed design is better than that of standard MTF. The SM operation property can be enhanced with the decreases of bending radius. The proposed design shows great potential in high power fiber lasers with compact structure.

## Introduction

Over the last decades, high power fiber lasers have developed rapidly due to their beam quality, heat dissipation, brightness, operating costs and efficiency [1–3]. However, with the further increase of output power, the nonlinear effect of fiber becomes the most important challenge. To eliminate the challenges induced by high power output, large mode area (LMA) fibers have become the preferred choice.

A large number of transverse modes always lead to the mode competition and instability of output [4, 5]. It is important for high power fiber lasers to achieve LMA and effective single-mode (SM) operation simultaneously. A series of LMA fibers have been proposed to achieve effective SM operation, such as double-clad fibers [6], low numerical aperture (NA) step-index fibers [7], chirally-coupled-core (CCC) fibers [8], photonic crystal fibers (PCF) [9], segmented cladding fibers (SCF) [10,11], gain-guided and index anti-guided (GG+IAG) optical fibers [12], microstructured fibers [13, 14], multilayer cladding fibers [15–17] and multi-trench fibers (MTFs) [18–20]. However, the application limits of these fibers are the complex and expensive fabrication and detrimental bending effects.

Rod MTFs can achieve large mode area and excellent high-order modes (HOMs) suppression capability [19]. However, when MTF is bent, the mode region must be less than 800 μm^2^ in order to maintain the HOMs suppression capability. The mode area is about 410 μm^2^ with 30 μm core diameter when bending radius is 20 cm. Sun et al. broke gaps on two outer trenches to improve the bending performance [21,22].

In this paper, we demonstrate that all trenches broken MTF can improve the SM operation outstandingly. The gap width can be adjusted to control the leakage losses of the fiber. The loss ratio between lowest-HOM and fundamental mode (FM) is more than 300 with the mode area of 840 μm^2^ under bending radius of 15 cm. The propagation characteristics with different fiber parameters are also discussed in detail.

## Optical fiber structure and theoretical model

**Fig 1:**
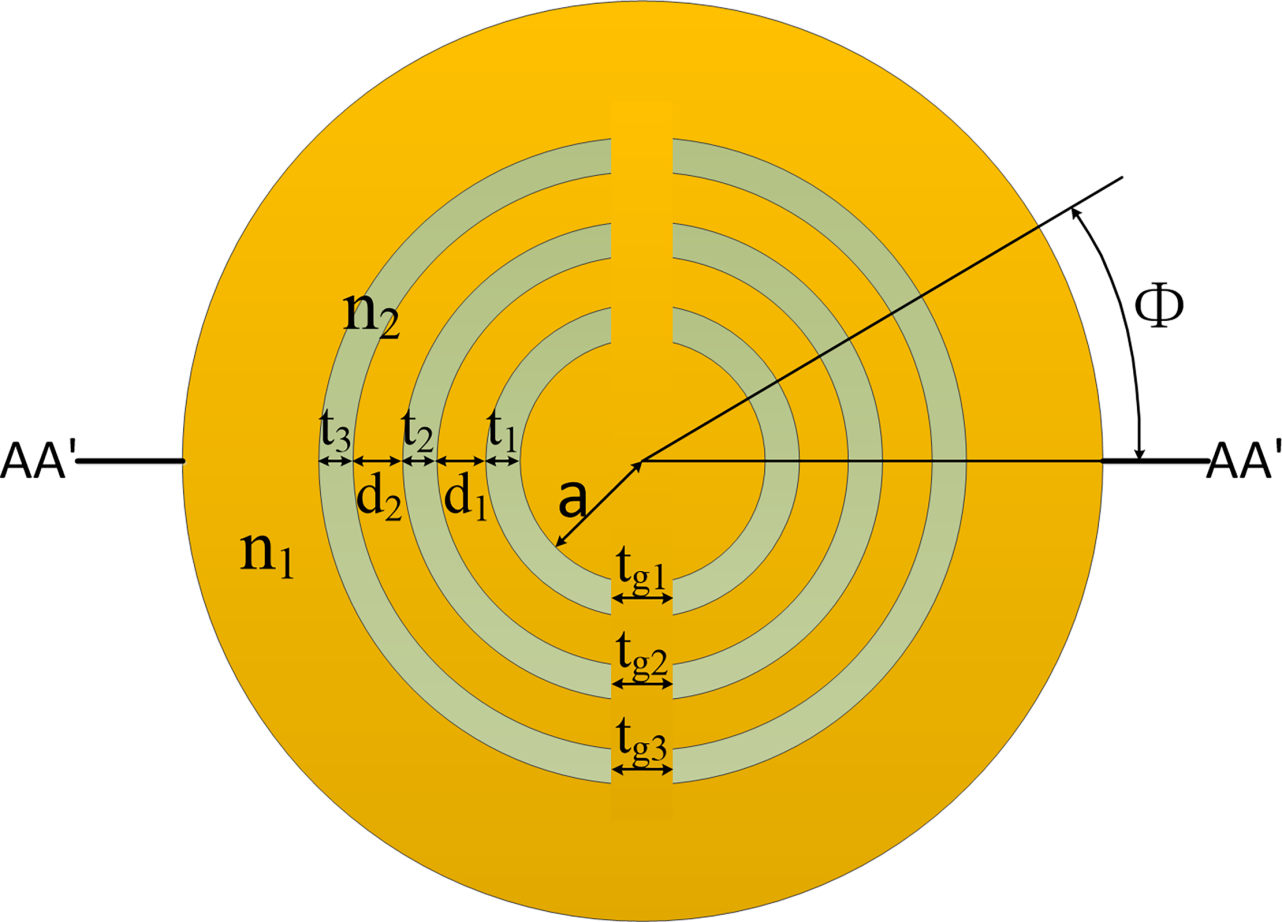
Cross section and notations of the proposed leaky-MTF with leakage gaps.

The proposed modified MTF structure is shown in Fig 1. The leaky-MTF can be fabricated by carving grooves in MTF and inserting rods into these grooves [21, 22]. The gray region represents the low refractive-index (RI) of *n*_*2*_ = 1.444 at the wavelength of 1064 nm. The yellow region represents the high RI (*n*_*1*_). The notations are also shown in Fig 1, where *a* stands for core radius. *t*_*1*_, *t*_*2*_ and *t*_*3*_ are the thickness of low RI trenches, respectively. *d*_*1*_ and *d*_*2*_ are the thickness of high RI rings, respectively. *Δn* = *n*_*1*_*—n*_*2*_ is the RI difference between the core and the low RI trenches. *t*_*g1*_, *t*_*g2*_ and *t*_*g3*_ are the gap width, respectively. *Φ* is the bending angle between the actual bending orientation and the reference bending orientation (*AA’*). It should be noted that, when we mention *t*, it represents all the low RI trenches (*t*_*1*_, *t*_*2*_ and *t*_*3*_). For example, when *t* = 3 μm, it represents *t*_*1*_ = *t*_*2*_ = *t*_*3*_ = 3 μm. For *t* ranges from 3–9 μm, it denotes *t*_*1*_, *t*_*2*_ and *t*_*3*_ range from 3–9 μm simultaneously. Similarily, *d* represents *d*_*1*_ and *d*_*2*_; *t*_*gap*_ represents *t*_*g1*_, *t*_*g2*_ and *t*_*g3*_.

The finite element method (FEM) is used in complex fiber structure analysis due to its high calculation precision. It is the most commonly used method in microstructure optical fiber simulation. The numerical simulations are calculated by using COMSOL Multiphysics software based on FEM, together with anisotropic perfectly matched layers (PMLs). For the proposed theoretical analysis of leaky-MTF, a 20-μm-thick circular PML is set outside the fiber cladding. Bending has an effect on the RI distribution of silica optical fiber. The bent fiber can be equivalent to a straight fiber through a proper mathematical transformation. After being modified with additional stress perturbations, the bent fiber RI distribution *nꞌ(x*,*y)* can be expressed as [23,24]:

$$n'(x,y)=n(x,y)\;*\;(1+\frac{\vec{x}\cos\;\Phi +\vec{y}\sin\;\Phi }{\rho *R})$$ (1)

where *n(x*,*y)* is the initial RI distribution of straight fiber, *R* is bending radius, *Φ* is the bending orientation angle (as shown in Fig 1) and *ρ* (here fixed to 1.25) is correction coefficient taking account of the stress factor.

Bending loss and mode area *A*_*eff*_ can be calculated by the following equations [25, 26]:

$$Loss=\frac{40\pi }{ln(10)\lambda }Im\left(n_{eff}\right)$$ (2)

$$A_{eff}=\frac{{\left(\iint \left|E^{2}\right|dxdy\right)}^{2}}{\iint {\left|E\right|}^{4}dxdy}$$ (3)

Where *n*_*eff*_ is the effective RI of modes, *E* is the electric field inside the fiber and *λ* is the operation wavelength, which is set as 1064 nm in this paper. Defaultly, the reference bending direction is *AA’* (*Φ* = 0°), if not specially mention. In practical applications, FM loss less than 0.1 dB/m and HOMs loss more than 1 dB/m is considered as the basic condition of effective SM operation [27].

The loss ratio (LR) means ratio between lowest-HOM and FM in fiber, which is defined as

$$LR=\frac{Loss(lowest-HOM)}{Loss(FM)}$$ (4)

Where *Loss (lowest-HOM)* is the loss of lowest-HOM and *Loss (FM)* is the loss of fundamental mode. In this paper, *Loss(LP*_*01*_*)* refers to the loss of LP_01_ mode, *Loss(LP*_*11v*_*)* refers to the loss of LP_11v_ mode, *Loss(LP*_*11h*_*)* refers to the loss of LP_11h_ mode.

## Method verification

**Fig 2:**
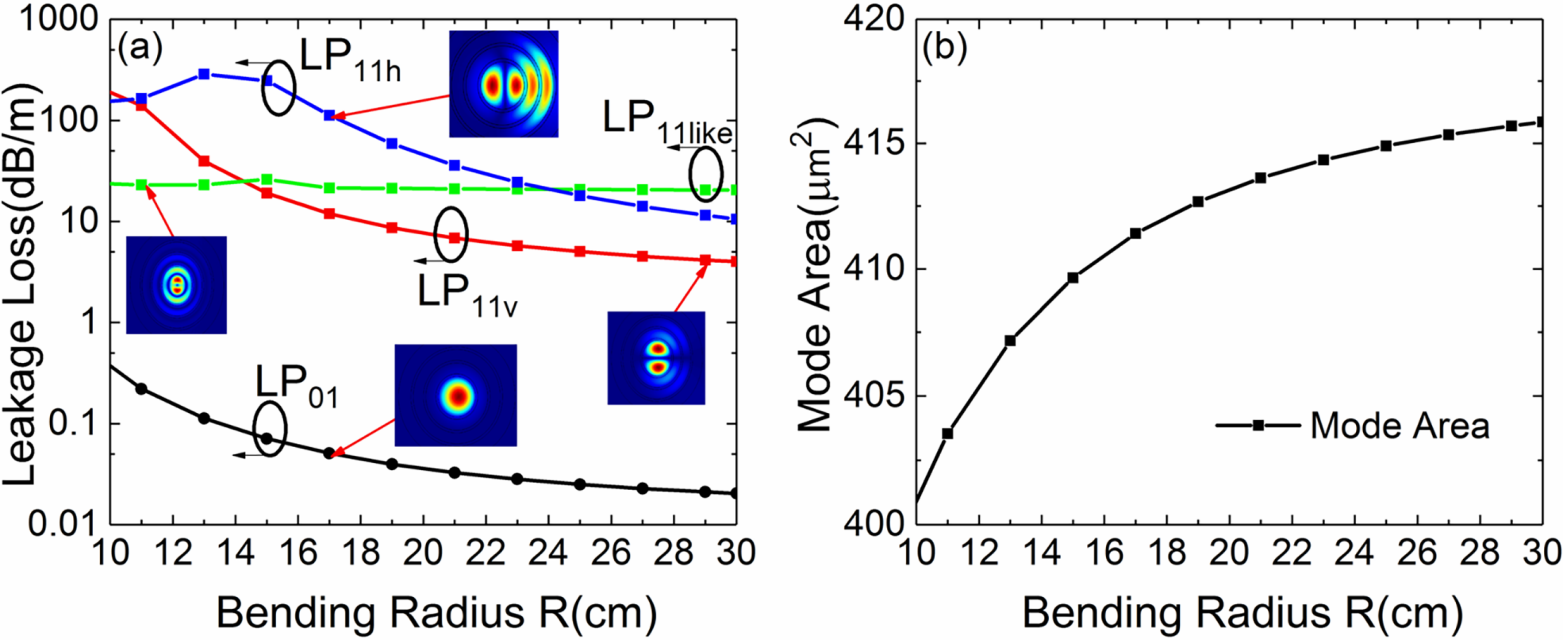
Fiber performance with different bending radius. (a) Simulated losses of the FM and HOMs of the standard MTF with *a* = 15 μm, *t* = 2 μm, *d* = 8 μm and *Δn* = 0.005 for different bend radii. Inset pictures show the computed normalized electric field of the FM and HOMs at a bend radius of 17 cm, 29 cm, 21 cm and 11 cm, respectively. (b) The *A*_*eff*_ for the FM at different bend radii.

In order to confirm the accuracy of the simulation method and contrast the difference with our design, we simulate the standard MTF firstly. The standard MTF with structural parameters *a* = 15 μm, *t* = 2 μm, *d* = 8 μm and *Δn* = 0.005 as that in [18] is simulated at wavelength of 1064 nm. Fig 2(A) shows the numerically simulated losses of the FM and HOMs of standard MTF as a function of bending radius. Fig 2(B) shows the simulated *A*_*eff*_ of the FM. The inset pictures show the simulated normalized electric field of LP_01_, LP_11v,_ LP_11h_ and LP_11like_ mode at a bending radius of 17 cm, 29 cm, 21 cm and 11 cm, respectively. When bending radius ranges from 10 cm to 14 cm, the lowest-HOM is LP_11like_ mode. When bending radius ranges from 14 cm to 30 cm, the lowest-HOM is LP_11_ mode. The *A*_*eff*_ remains larger than 400 μm^2^ when bending radius ranges from 10–30 cm. The computed data are similar to the results given in Ref.18. Therefore, the accuracy of the simulation method in this paper is reliable.

It can be seen from Fig 2(A) that, LP_11v_ and LP_11h_ mode are separated because of the birefringence caused by bending. LP_11h_ mode suffers more leakage loss than LP_11v_ mode. We propose leaky-MTF by breaking two gaps up and down on each low RI trench, as shown in Fig 1. The effects of gaps are shown in the following chapters.

## Numerical simulations

### Effects of gaps

**Fig 3:**
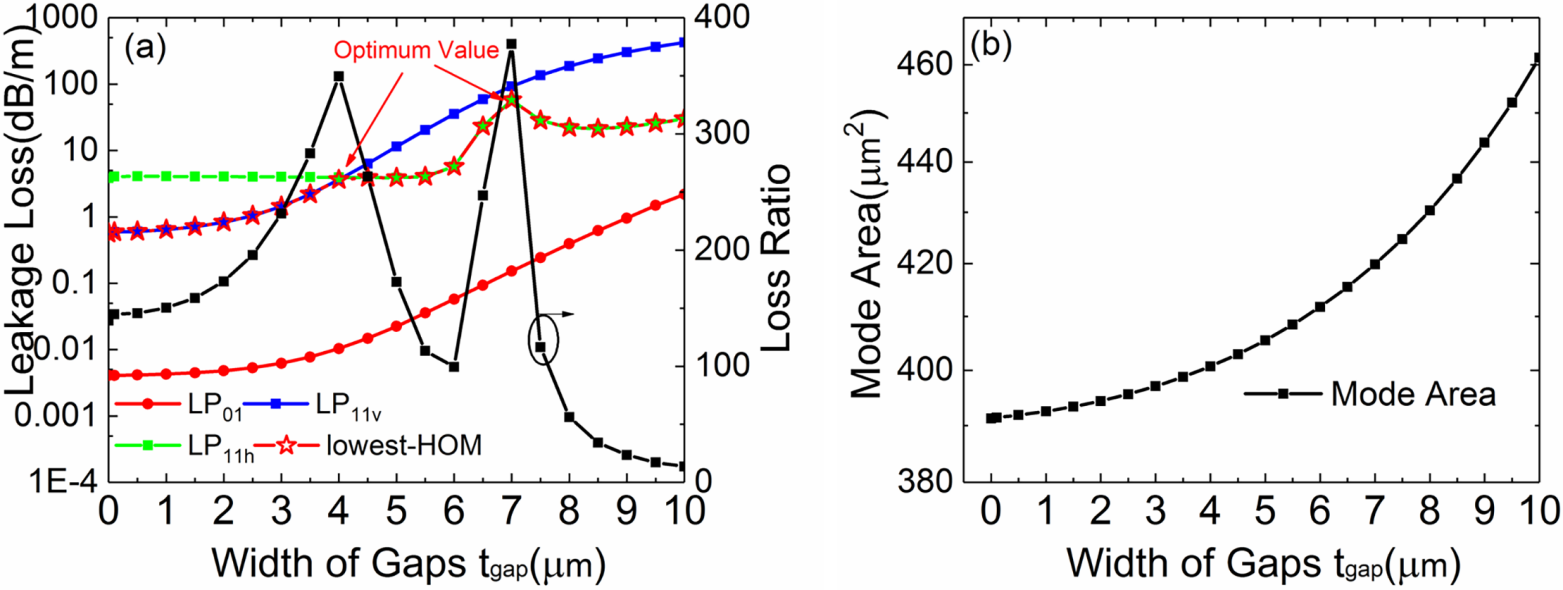
Fiber performance with different width of gaps. (a) Simulated losses of the FM and HOMs and LR of the leaky-MTF with *a* = 15 μm, *t* = 2 μm, *d* = 8 μm and *Δn* = 0.005 for different bend radii. (b) The *A*_*eff*_ for the FM at different bend radii.

**Fig 4:**
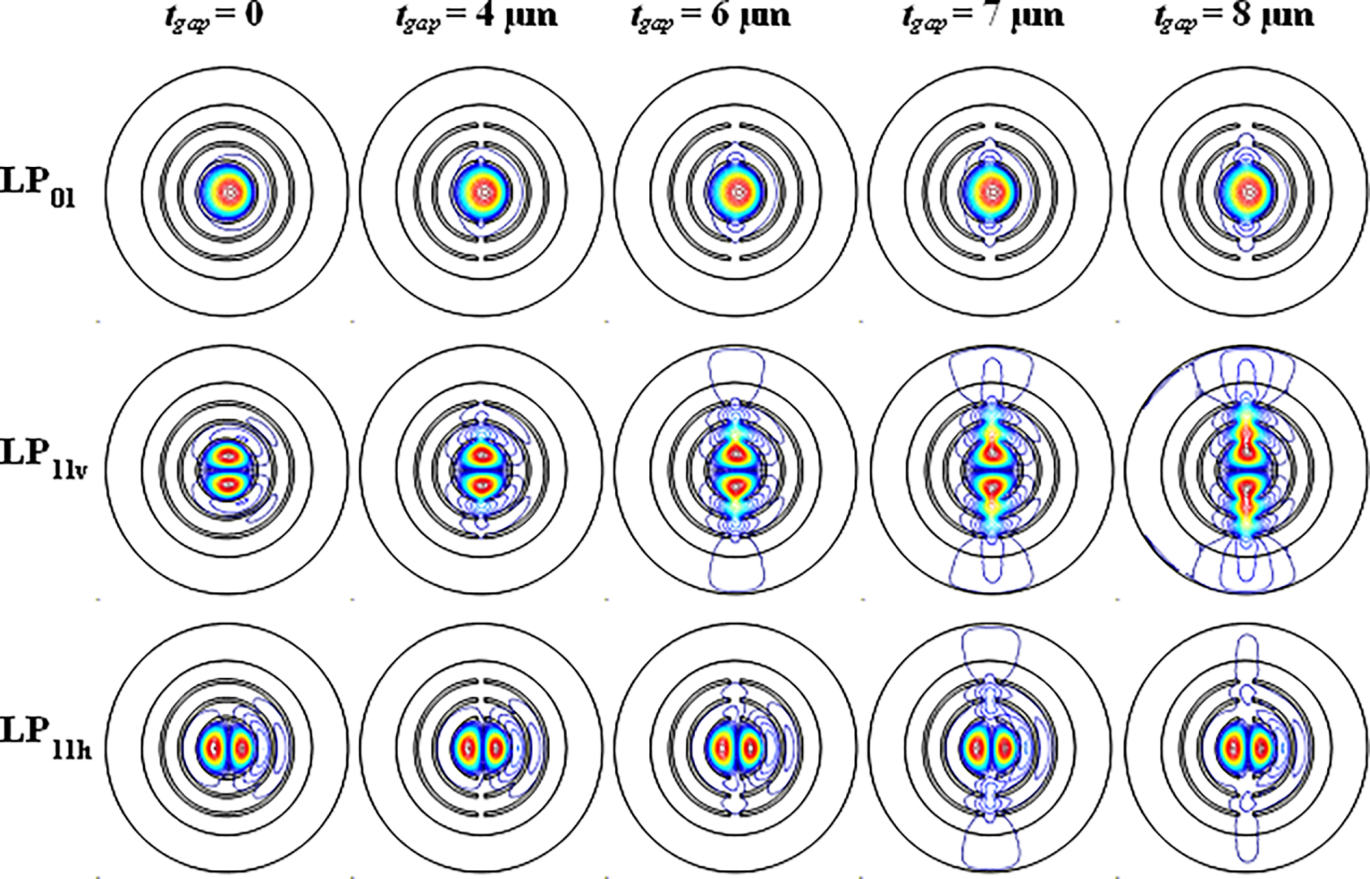
Contour line graphs of the mode field distribution of LP_01_, LP_11v_ and LP_11h_ mode of the leaky-MTF with *t*_*gap*_ = 0, 4 μm, 6 μm, 7 μm and 8 μm.

Fig 3(A) shows the leakage loss of LP_01_, LP_11v_, LP_11h_ and lowest-HOMs for different gap width. The other fiber parameters are the same as Fig 2. The LR is also plotted in Fig 3(A) (right axis). The *A*_*eff*_ is shown in Fig 3(B). The losses of LP_01_ and LP_11v_ mode increase when t_gap_ enlarges. The loss of LP_11h_ mode remains stable when *t*_*gap*_ changes from 0–6 μm but increases when *t*_*gap*_ is larger than 6 μm. To illustrate this case, the contour line graphs of the mode field distribution of three modes with *t*_*gap*_ = 0 μm, 4 μm, 6 μm, 7 μm and 8 μm are shown in Fig 4. LP_11h_ mode remains stable when *t*_*gap*_ changes from 0–6 μm because the mode distribution is far from gap. At *t*_*gap*_ = 4 μm, the mode leakage of LP_11v_ mode is equal to LP_11h_ mode (*Loss(LP*_*11v*_*) = Loss(LP*_*11h*_*)*). It is the first peak value for lowest-HOMs. When *t*_*gap*_ is larger than 6 μm, the mode distribution is close to gap and the leakage of LP_11h_ mode increase. However, At *t*_*gap*_ = 7 μm, *Loss(LP*_*11h*_*)* has the peak value because the leakage and resonance reach the largest. It is the second peak value for lowest-HOMs. Corresponding with lowest-HOMs, it has optimum values at *t*_*gap*_ = 4 μm and 7 μm.

For standard MTF (*t*_*gap*_ = 0), the loss of LP_01_, LP_11v_ and LP_11h_ mode is 0.004, 0.56 and 3.8 dB/m, respectively. For *t*_*gap*_ = 4 μm, the loss of LP_01_, LP_11v_ and LP_11h_ mode is 0.01, 3.6 and 3.9 dB/m, respectively. The LR increases by 150% from 139 to 350. For *t*_*gap*_ = 7 μm, the loss of LP_01_, LP_11v_ and LP_11h_ mode is 0.15, 92 and 57 dB/m, respectively. The LR increases by 170% from 139 to 377. The introduction of gaps has an excellent improvement for SM operation. As shown in Fig 3, to achieve a high differential loss factor, a proper gap width is necessary. As the *t*_*gap*_ increases, the *A*_*eff*_ increases from 390 μm^2^ to 460 μm^2^.

**Fig 5:**
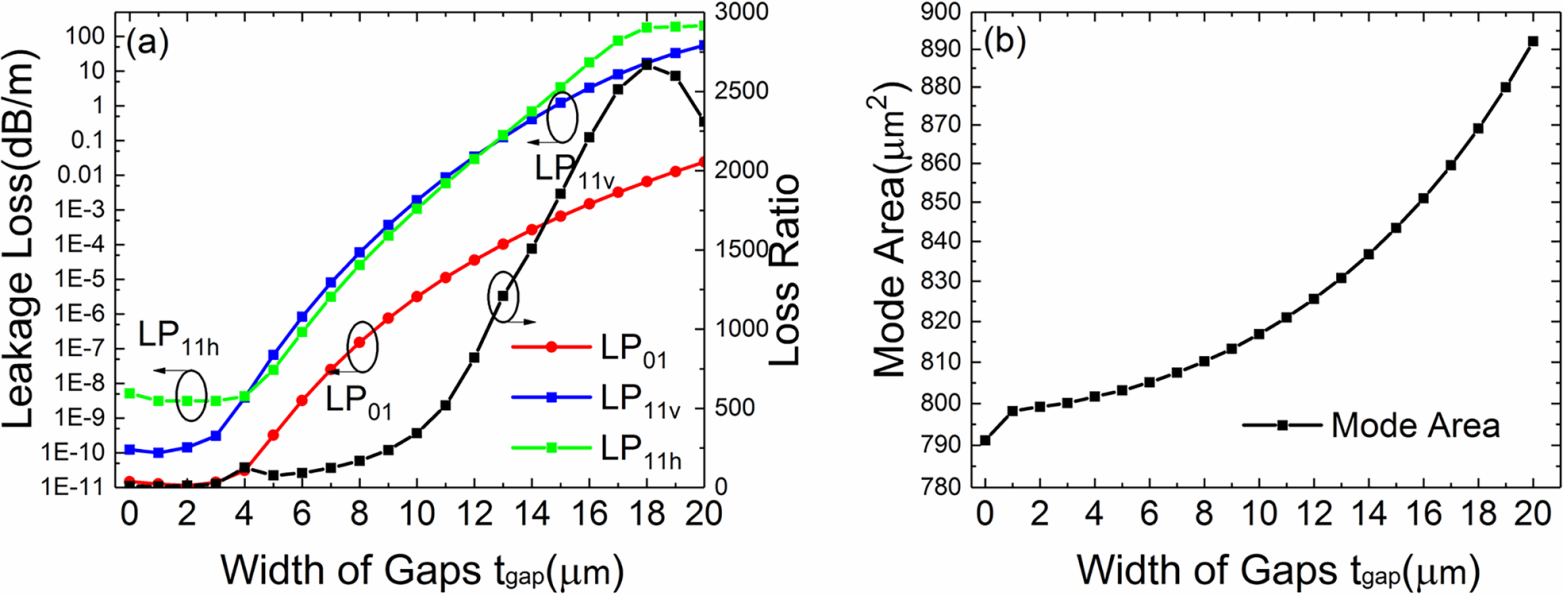
Fiber performance with different width of gaps. (a) Simulated losses of the FM and HOMs and LR of the leaky-MTF with *a* = 25 μm, *t* = 6 μm, *d* = 10 μm, *R* = 15 cm and *Δn* = 0.007 for different gap width. (b) The *A*_*eff*_ for the FM at different gap width.

**Fig 6:**
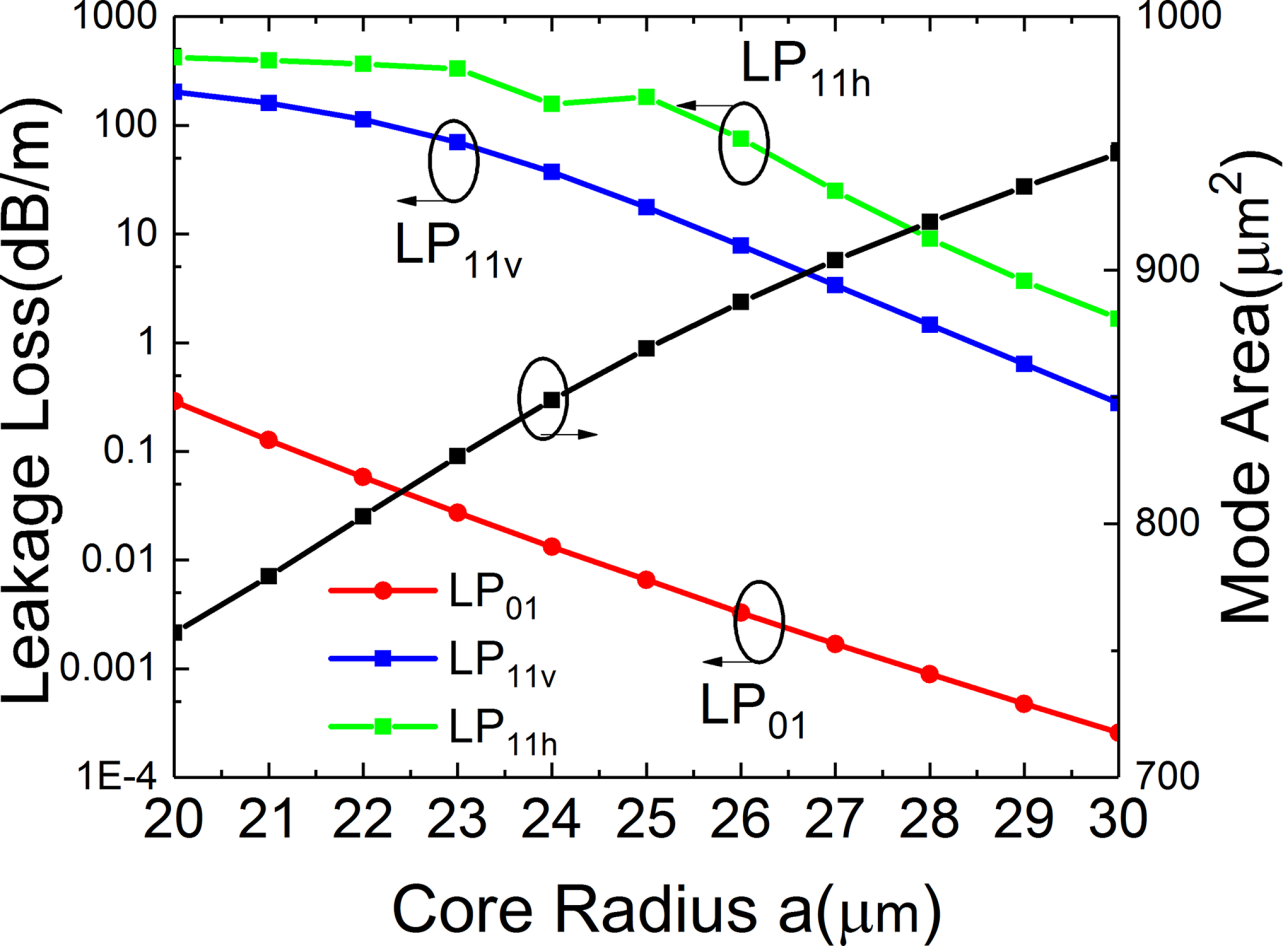
Simulated losses of the FM and HOMs and mode area of FM of the leaky-MTF with *t*_*gap*_ = 18 μm, *t* = 6 μm, *d* = 10 μm, *R* = 15 cm and *Δn* = 0.007 for different core radius.

**Fig 7:**
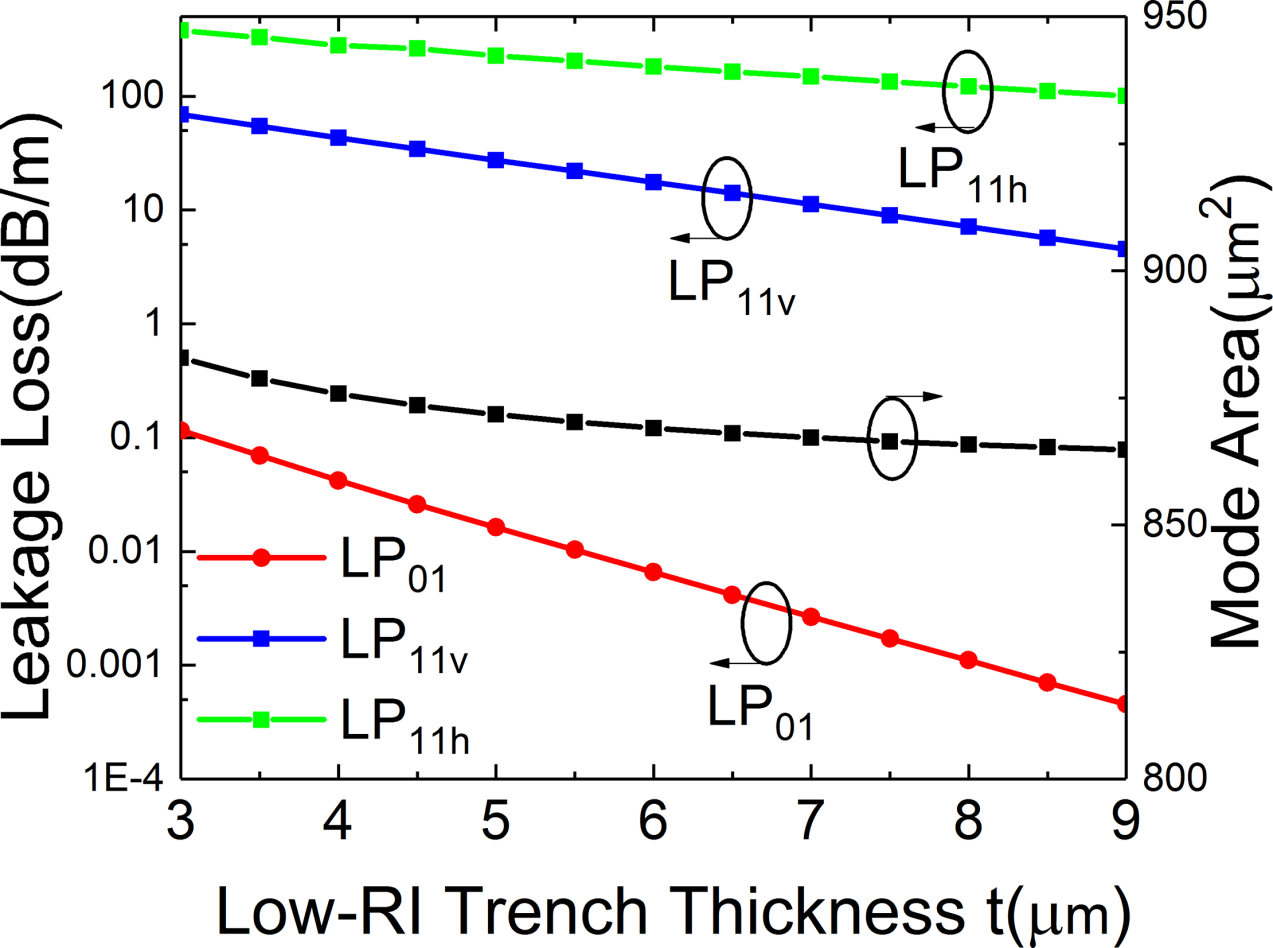
Simulated losses of the FM and HOMs and mode area of FM of the leaky-MTF with *a* = 25 μm, *t*_*gap*_ = 18 μm, *d* = 10 μm, *R* = 15 cm and *Δn* = 0.007 for different thickness of low RI trenches.

**Fig 8:**
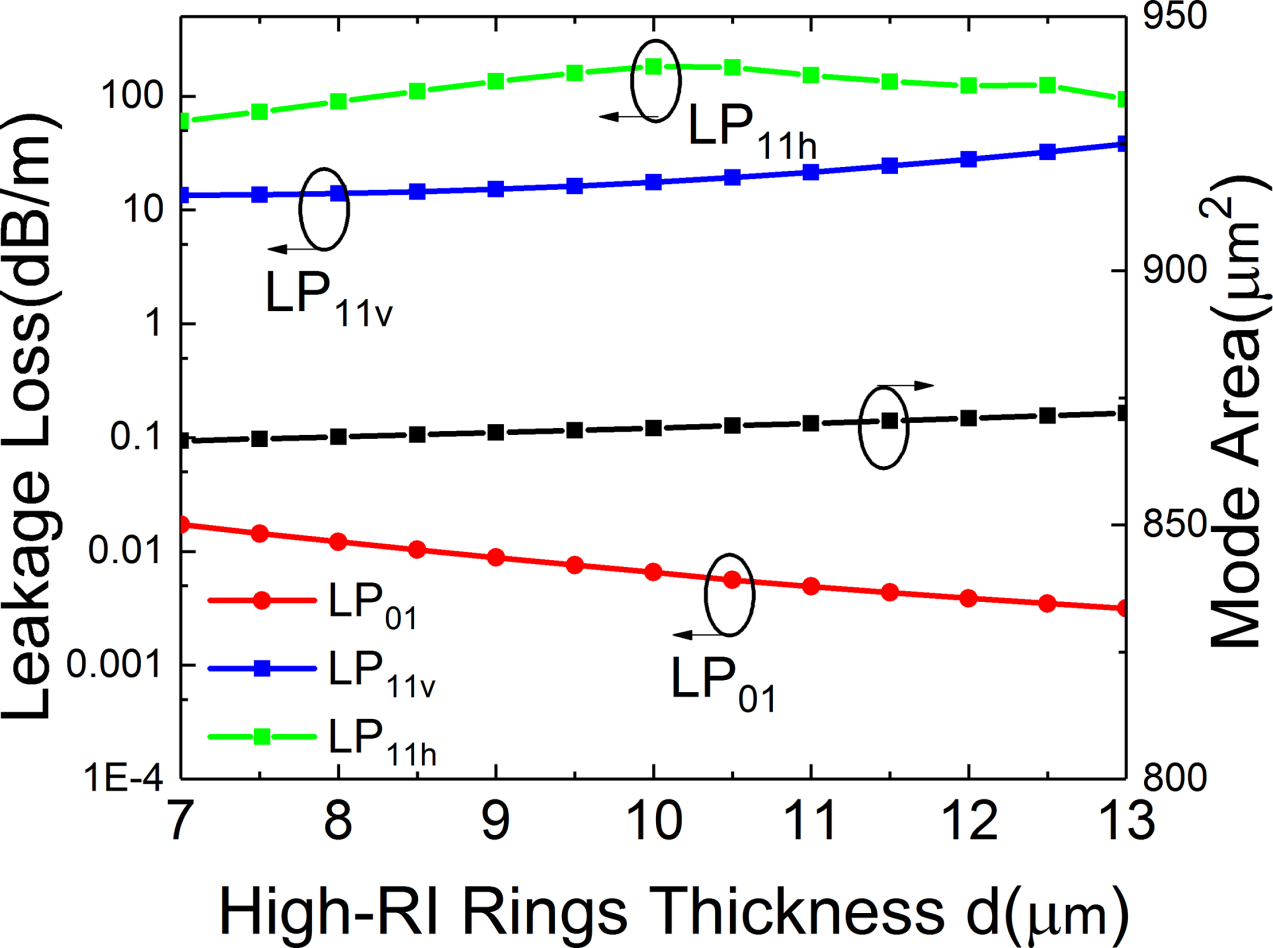
Simulated losses of the FM and HOMs and mode area of FM of the leaky-MTF with *a* = 25 μm, *t* = 6 μm, *t*_*gap*_ = 18 μm, *R* = 15 cm and *Δn* = 0.007 for different thickness of high RI rings.

**Fig 9:**
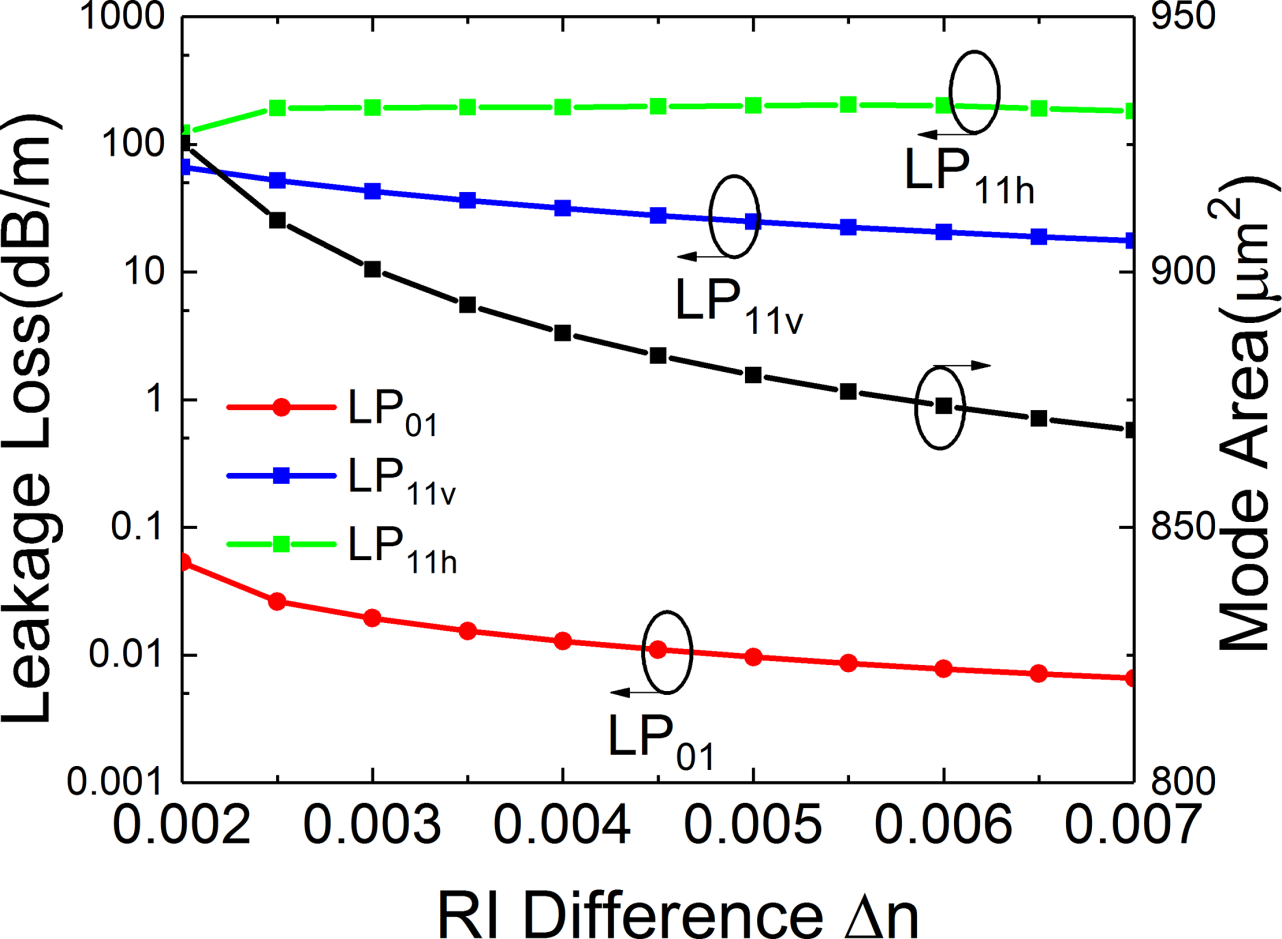
Simulated losses of the FM and HOMs and mode area of FM of the leaky-MTF with *a* = 25 μm, *t*_*gap*_ = 18 μm, *t* = 6 μm, *d* = 10 μm and *R* = 15 cm for different RI difference.

**Fig 10:**
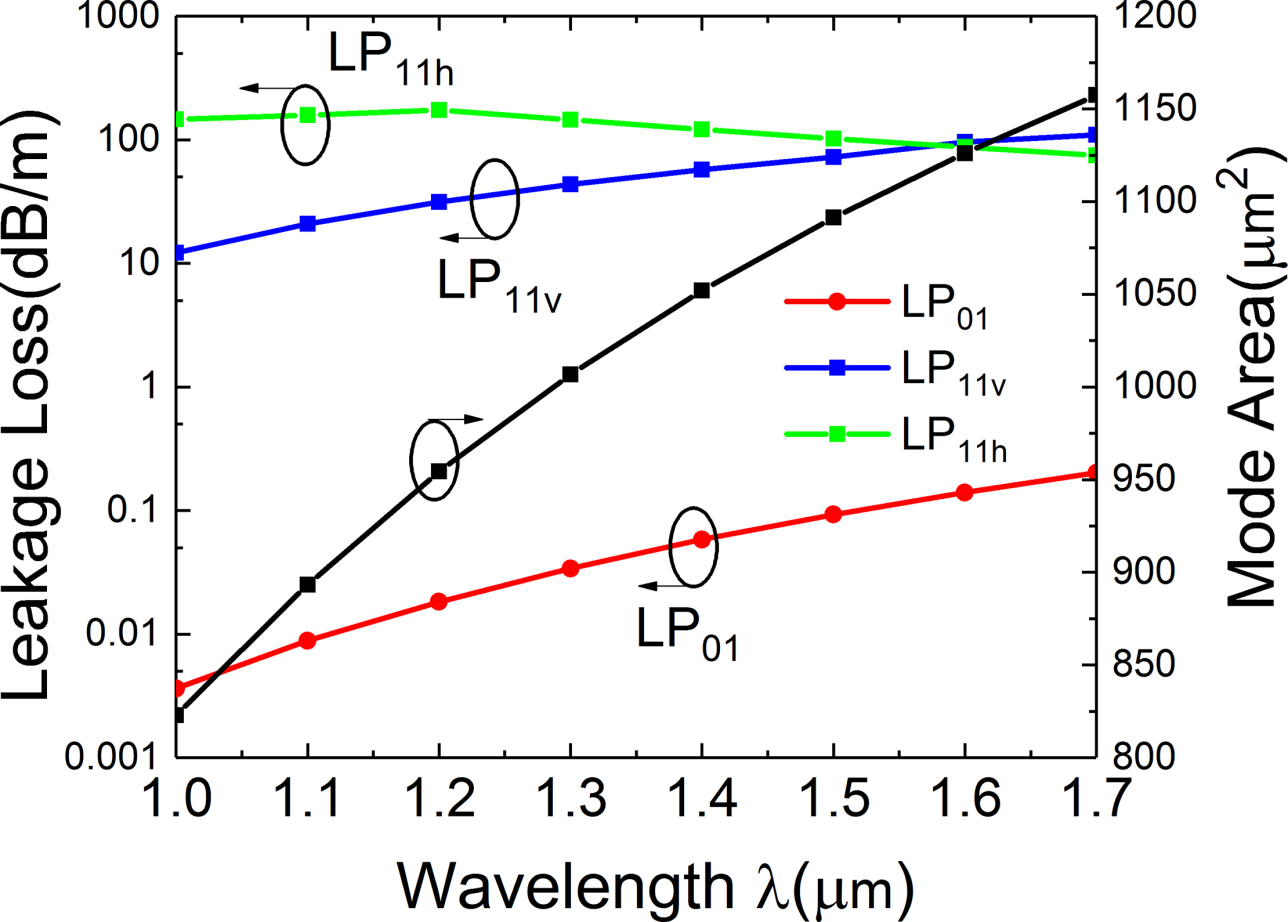
Simulated losses of the FM and HOMs and mode area of FM of the leaky-MTF with *a* = 25 μm, *t*_*gap*_ = 18 μm, *t* = 6 μm, *d* = 10 μm, *R* = 15 cm and *Δn* = 0.007 for different wavelength.

**Fig 11:**
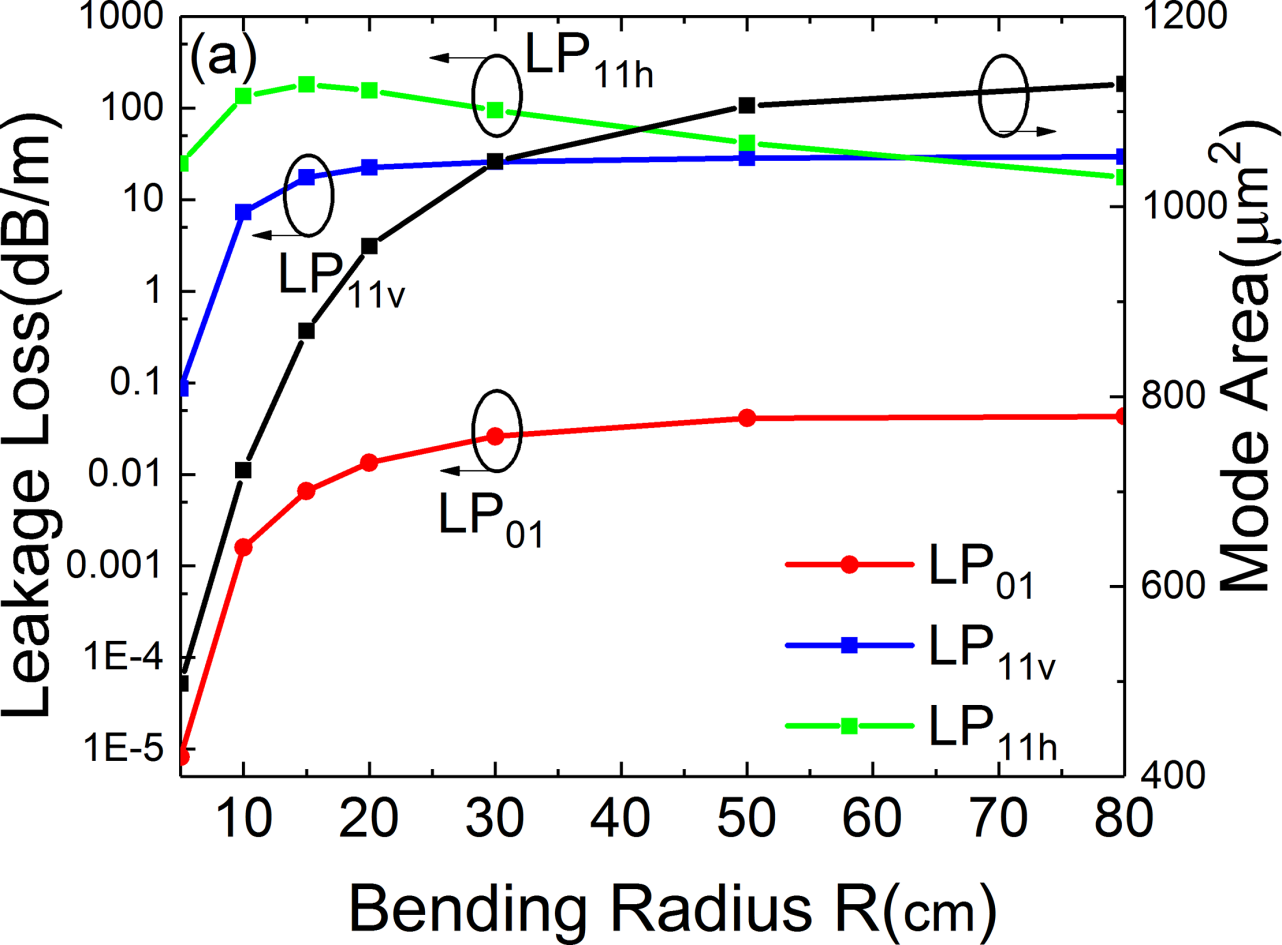
Simulated losses of the FM and HOMs and mode area of FM of the leaky-MTF with *a* = 25 μm, *t* = 6 μm, *d* = 10 μm, *t*_*gap*_ = 18 μm, *Δn* = 0.007 and *Φ* = 0° for different bending radius.

**Fig 12:**
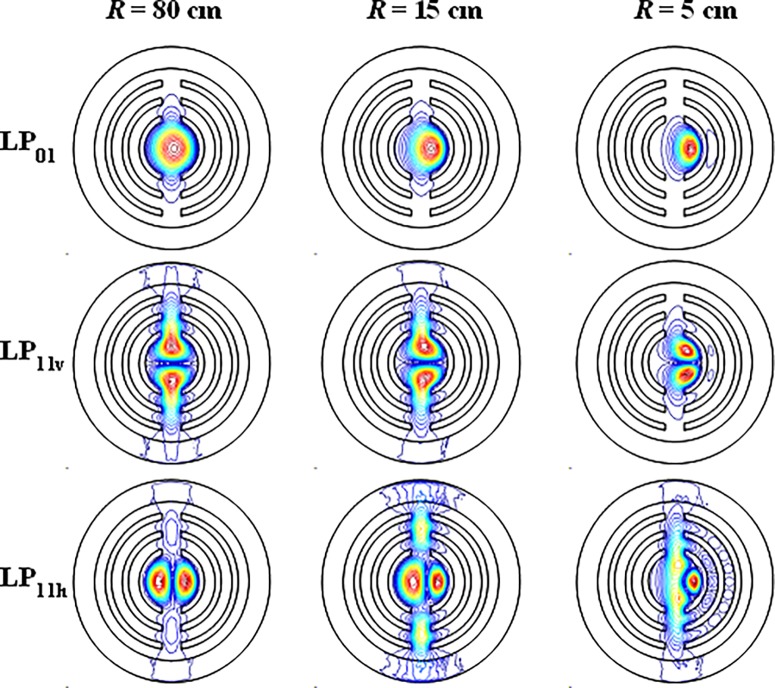
Contour line graphs of the mode field distribution of LP_01_, LP_11v_ and LP_11h_ mode of the leaky-MTF with *R* = 80 cm, 15 cm and 5 cm.

**Fig 13:**
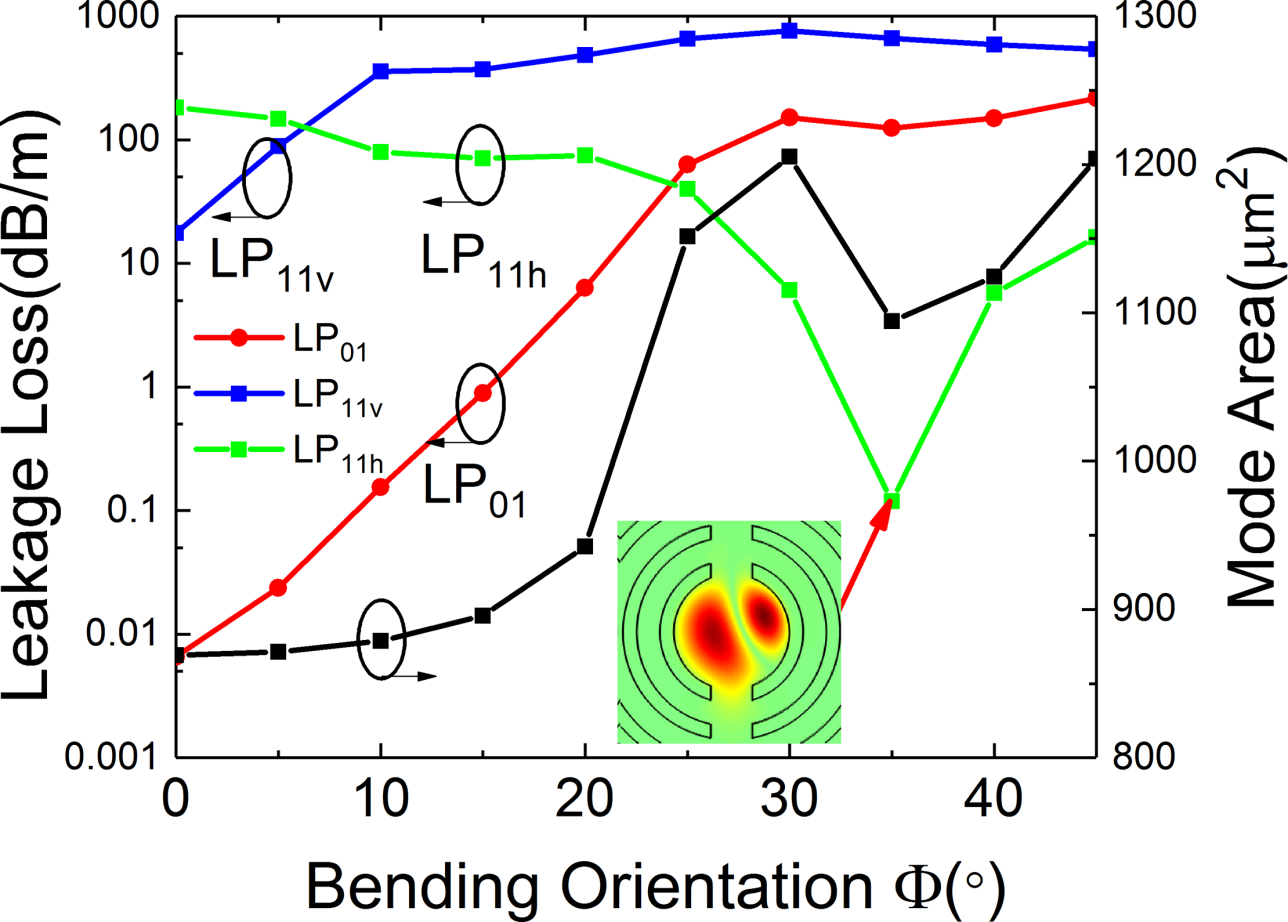
Simulated losses of the FM and HOMs and mode area of FM of the leaky-MTF with *a* = 25 μm, *t*_*gap*_ = 18 μm, *t* = 6 μm, *d* = 10 μm, *R* = 15 cm and *Δn* = 0.007 for different bending orientation.

**Fig 14:**
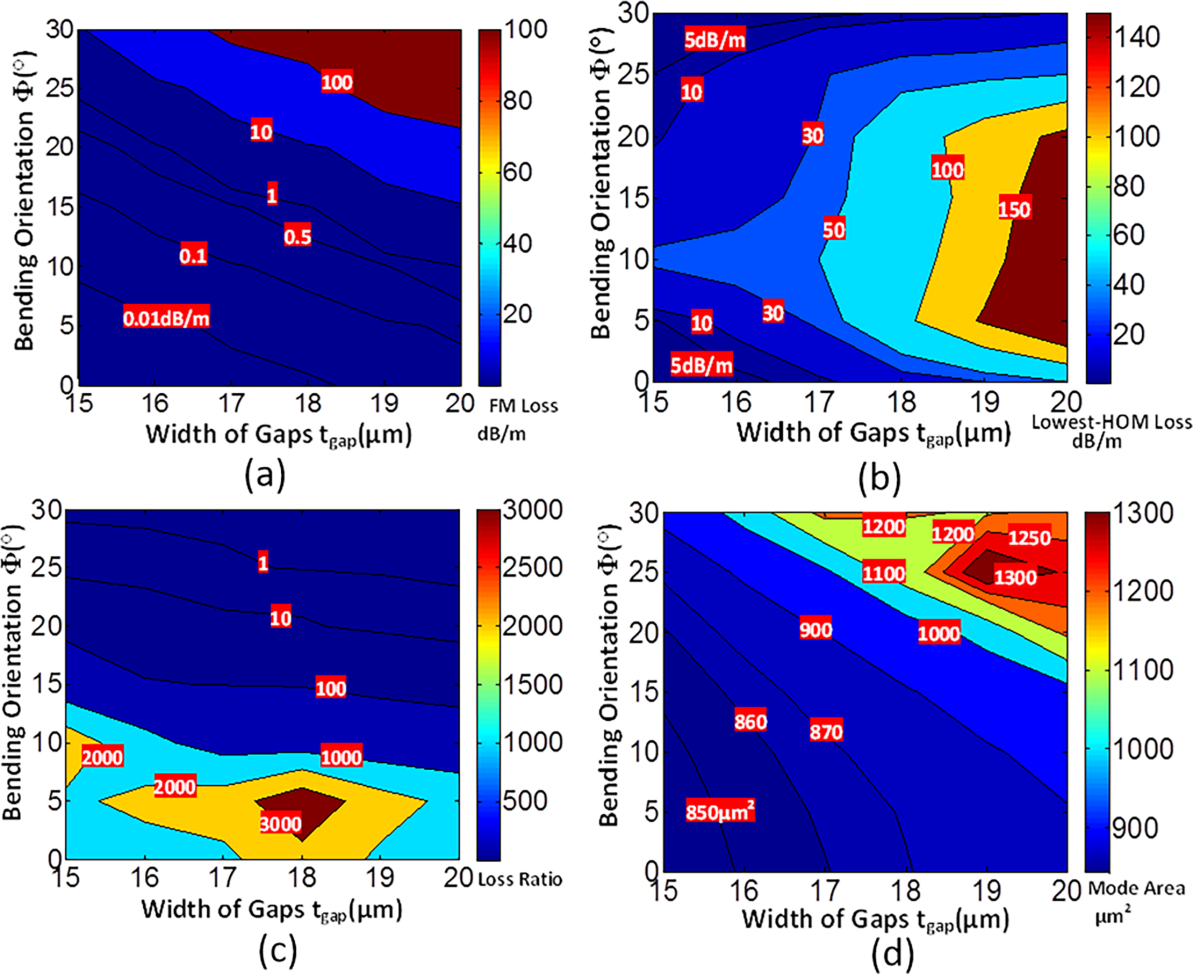
**Joint effects of bending orientation (*Φ*) and gap width (*t***_***gap***_**)** on (a) loss of FM, (b) loss of lowest-HOMs, (c) LR and (d) mode area with *a* = 25 μm, *t* = 6 μm, *d* = 10 μm, *R* = 15 cm and *Δn* = 0.007.

**Fig 15:**
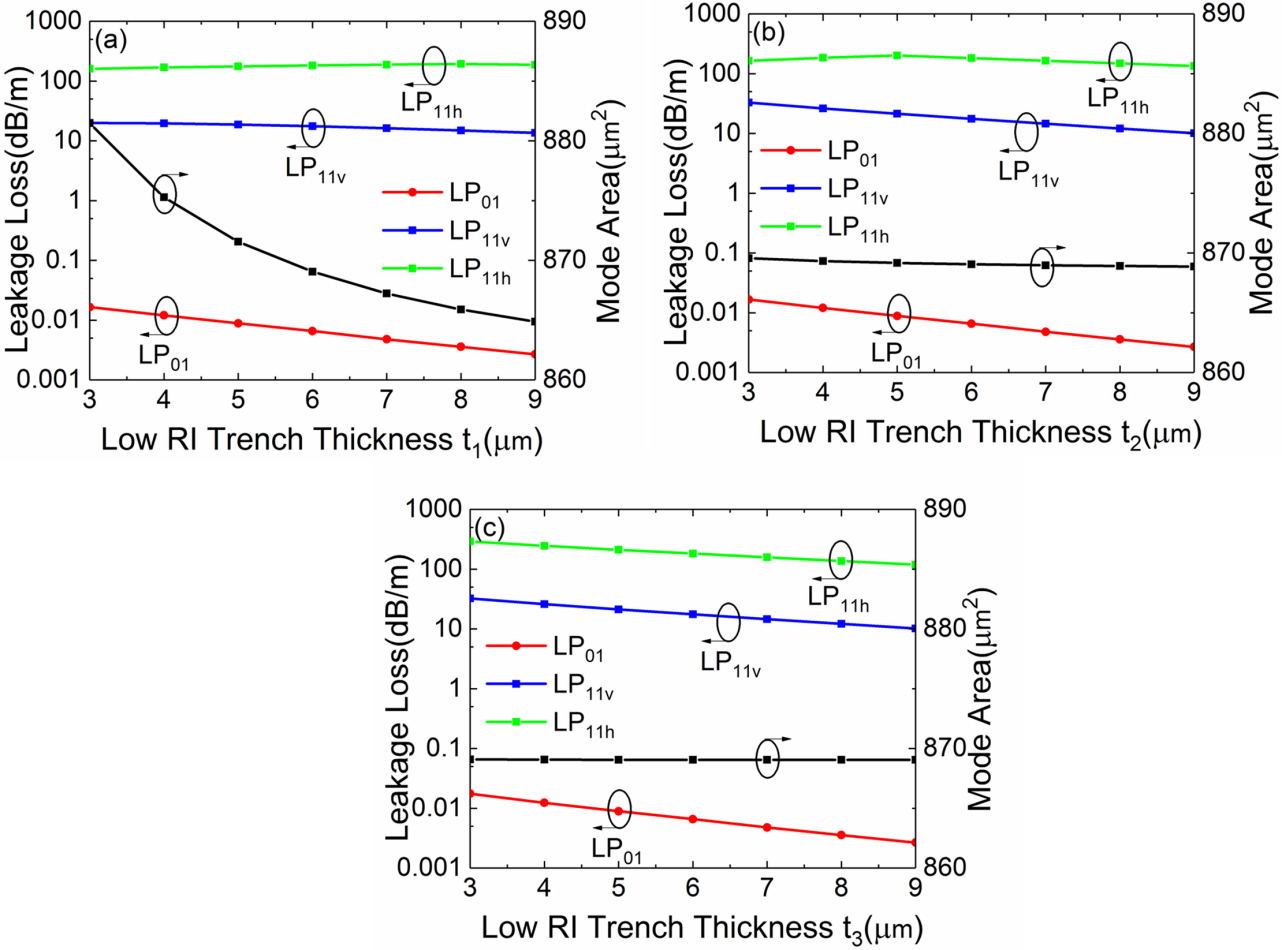
(a-c) Simulated losses of the FM and HOMs and mode area of FM of the leaky-MTF with *a* = 25 μm, *t*_*gap*_ = 18 μm, *t* = 6 μm, *d* = 10 μm, *R* = 15 cm and *Δn* = 0.007 for different *t*_*1*_, *t*_*2*_ and *t*_*3*_, respectively.

**Fig 16:**
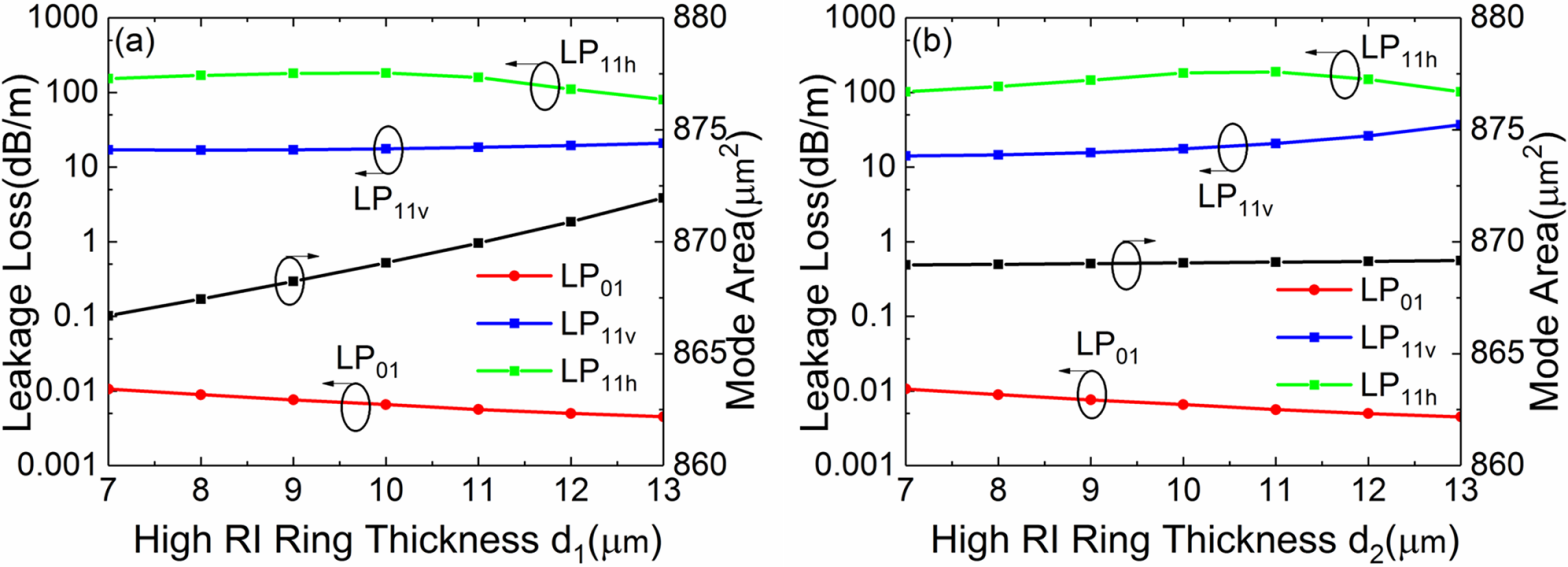
(a-b) Simulated losses of the FM and HOMs and mode area of FM of the leaky-MTF with *a* = 25 μm, *t*_*gap*_ = 18 μm, *t* = 6 μm, *d* = 10 μm, *R* = 15 cm and *Δn* = 0.007 for different *d*_*1*_ and *d*_*2*_, respectively.

**Fig 17:**
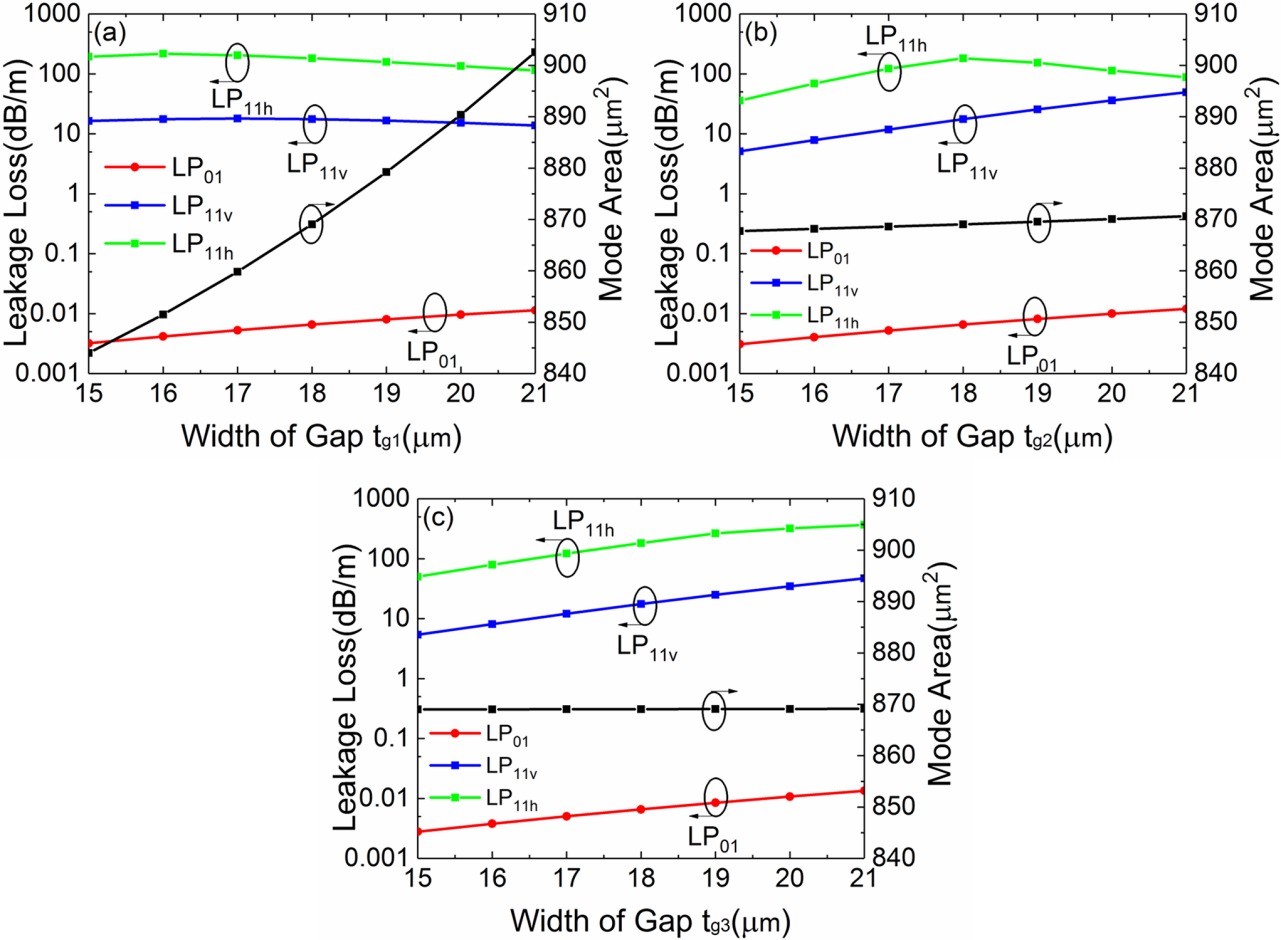
(a-c) Simulated losses of the FM and HOMs and mode area of FM of the leaky-MTF with *a* = 25 μm, *t*_*gap*_ = 18 μm, *t* = 6 μm, *d* = 10 μm, *R* = 15 cm and *Δn* = 0.007 for different *t*_*g1*_, *t*_*g2*_ and *t*_*g3*_, respectively.

In order to further enlarge the effective mode area, we choose the parameters of fiber as *a* = 25 μm, *t* = 6 μm, *d* = 10 μm, *t*_*gap*_ = 18 μm, *Δn* = 0.007 and *R* = 15 cm. The effects of various parameters of the structure are studied and summarized in Figs 5–17. Fig 5(A) shows the effect of the gap width on the loss of LP_01_, LP_11v_ and LP_11h_ modes and LR. Fig 5(B) shows the effective mode are of FM. The variation trend of loss curves and LR curve are similar to Fig 3. The loss of LP_01_ and LP_11v_ mode increase with the increase of gaps. The loss of LP_11h_ mode keeps stable at first, and then increases. It can be seen from Fig 5(A) that, the LR has two peak values at *t*_*gap*_ ≈ 4 μm and *t*_*gap*_ ≈ 18 μm. For standard MTF (*t*_*gap*_ = 0), the loss of LP_01_, LP_11v_ and LP_11h_ mode are 1.5×10^−11^, 1.2×10^−10^ and 5×10^−9^ dB/m, respectively. The LR is 8.4 and the mode area of FM is 791 μm^2^. At *t*_*gap*_ = 4 μm, the loss of LP_01_, LP_11v_ and LP_11h_ mode are 3×10^−11^, 3.9×10^−9^ and 4.2×10^−9^ dB/m, respectively. The LR is 126 which arise by 1400% and the mode area of FM is 801 μm^2^. At *t*_*gap*_ = 18 μm, the loss of LP_01_, LP_11v_ and LP_11h_ mode are 0.006, 17 and 182 dB/m, respectively. The LR is 2667 which arises by 31600% theoretically and the mode area of FM is 869 μm^2^.

It is clear that, gap can enlarge the core modes’ leakage losses thus it allows short fiber length to trip off HOMs. Meanwhile, LR can be tuned by adjusting to the gap width. By considering the largest loss of LP_11_ mode and LR, we choose *t*_*gap*_ = 18 μm.

### Effects of core radius

Fig 6 shows the variation of bending losses of the first three modes of fiber (LP_01_, LP_11v_ and LP_11h_) and *A*_*eff*_ of FM on core radius (*a*) of the proposed structure. When core radius increases, the losses of LP_01_, LP_11v_ and LP_11h_ mode decrease, while *A*_*eff*_ increases. By considering the trade-off between bending loss and mode area, we choose *a* = 25 μm to achieve both LMA and effective SM operation. In this case, *Loss (LP*_*01*_*)* < 0.01 dB/m and *Loss (LP11)* > 0.01 dB/m. Meanwhile, the *A*_*eff*_ = 869 μm^2^.

### Effects of low RI trenches

Fig 7 shows the effect of low RI trench thickness (*t*) on SM operation and *A*_*eff*_ of FM of the structure. It can be observed that the losses of LP_11v_ and LP_11h_ mode decrease slowly when *t* increases and the LP_01_ mode decreases sharply. The *A*_*eff*_ of FM also decreases when *t* increases. When *t* is in the range from 3.5 to 9 μm, the highest bending loss of LP_01_ mode is lower than 0.1 dB/m and the lowest bending loss for LP_11_ mode is higher than 4 dB/m, which is considered viable for SM operation. Meanwhile, the *A*_*eff*_ is larger than 850 μm^2^.

### Effects of high RI rings

Fig 8 illustrates the loss of LP_01_, LP_11v_ and LP_11h_ and *A*_*eff*_ of FM under different *d*. It can be observed that the bending loss of LP_01_ mode decreases when *d* increases. The loss of LP_11v_ mode and *A*_*eff*_ of FM have a slight increase when *d* increases. When *d* is in the range from 7 to 13 μm, the highest loss of LP_01_ mode is lower than 0.02 dB/m and the lowest loss of LP_11_ mode is large than 13 dB/m. Meanwhile, the *A*_*eff*_ ranges from 866 to 972 μm^2^.

### Effects of RI difference

The effects of the RI difference (*Δn*) are shown in Fig 9. The structural parameters are *a* = 25 μm, *t*_*gap*_ = 18 μm, *t* = 6 μm, *d* = 10 μm and *R* = 15 cm. Fig 9 illustrates leakage losses of LP_01_, LP_11v_ and LP_11h_ mode and *A*_*eff*_ of FM. From Fig 9, it can be seen that *Δn* has a slight effect on LP_01_, LP_11v_ and LP_11h_ mode and a serious effect on *A*_*eff*_ of FM. When *Δn* is in the range from 0.2 to 0.7, the highest loss of LP_01_ mode is lower than 0.06 dB/m and the lowest bending loss for LP_11_ mode is higher than 17 dB/m. It is considered that the bending loss conforms effective SM operation. The *A*_*eff*_ decreases from 925 to 869 μm^2^.

### Effects of wavelength

The effect of the wavelength (*λ*) have been investigated and presented in Fig 10. The *A*_*eff*_ of FM and bending losses of LP_01_ and LP_11v_ modes increase with the increases of *λ*. When *λ* is in the range from 1 to 1.7 μm, the highest loss of LP_01_ mode is lower than 0.2 dB/m and the lowest bending loss of LP_11_ mode is higher than 12 dB/m. The *A*_*eff*_ of FM increases from 822 to 1157 μm^2^. The fiber can achieve effective SM operation and LMA in a wide transmission bandwidth from 1 μm to 1.7 μm.

### Effects of bending

The effects of bending are shown in Figs 11–13. Fig 11 illustrates the leakage losses of LP_01_, LP_11v_ and LP_11h_ and mode area of FM at varying bending radius. The structural parameters are *a* = 25 μm, *t* = 6 μm, *d* = 10 μm, *t*_*gap*_ = 18 μm, *Δn* = 0.007 and *Φ* = 0°. When the bending radius ranges from 10 cm to 80 cm, the loss of LP_01_ is less than 0.05 dB/m and the loss of LP_11_ is larger than 5 dB/m. The *A*_*eff*_ ranges from 700 to 1130 μm^2^.

Fig 12 shows the contour line graphs of the mode field distributions of LP_01_, LP_11v_ and LP_11h_ modes of the leaky-MTF with *R* = 80 cm, 15 cm and 5 cm. *Loss (LP*_*11h*_*)* increases when R rangs from 80 cm to 5 cm. However, when the bending radius decreases, the losses of LP_01_ and LP_11v_ mode decrease. When *R* = 80 cm, the LP_01_ mode leaks from the gaps. When *R* = 5 cm, the mode distribution concentrates to the right side and the leakage loss reduced. So *Loss (LP*_*01*_*)* decreases with reducing bending radius. It is the same for *Loss (LP*_*11v*_*)*. When *R* = 80 cm, LP_11v_ mode suffers large loss because the mode leaks from gaps. LP_11v_ mode’s loss leaks a lot to the fiber cladding. When R decreases, at *R* = 15 cm, the leaked energy decreases. When *R* = 5 cm, the mode moves to the right and less energy leaks to the cladding.

Since the gap breaks the circular symmetry of MTF, the discussion of bending direction is necessary. Fig 13 illustrates leakage losses of LP_01_ LP_11v_ and LP_11h_ modes and mode area of FM at varying bending orientation. When the bending orientation increases from 0 to 45°, *Loss (LP*_*01*_*)* and *Loss (LP*_*11v*_*)* increases. However, *Loss (LP*_*11h*_*)* decreases and reaches a minimum value at *Φ* = 35°. The inset picture shows the field distribution of LP_11h_ mode at *Φ* = 35°. The mode area of FM has an obvious increase when bending orientation ranges from 0 to 30°. Moreover, the effect of *Φ* on the performance is analyzed further, because *Φ* is a critical parameter when the fiber is bended.

### Joint effects of gap and bending orientation

The joint effects of bending orientation and gap width are shown in Fig 14. The loss of FM, lowest-HOM, LR and mode area are plotted in Fig 14A–14D, respectively. Fig 14(A) shows that, the loss of FM increases with the increase of bending orientation and gap width. Fig 14(B) shows that, the lowest loss of HOMs increases with the increases of gap width. However, the lowest loss of HOMs increases when *Φ* ranges from 0 to 10°. Then it decreases when *Φ* ranges from 10° to 30°. It is because that, *Loss(LP*_*11v*_*)* is smaller than *Loss(LP*_*11h*_*)* with small bending orientation, while larger than *Loss(LP*_*11h*_*)* with large bending orientation. The lowest loss of HOMs increases and then decreases with bending orientation enlarges. It is obvious from Fig 13(C) that, the LR is large when the bending orientation is small. The LR increases with the increases of gap width. However, it has a maximum value with a proper gap width (at *t*_*gap*_ = 18 μm). The characteristic is corresponding with the conclusion obtained from Fig 4. It can be seen that, when *Φ* is less than 10° and *t*_*gap*_ ranges from 15 to 20 μm, the LR is larger than 100, which indicates that it conforms SM operation conditions. The *A*_*eff*_ increases when *t*_*gap*_ and *Φ* increase. It is because the leakage of FM is easier with greater bending and larger gap.

### Effects of one parameter changes

The discussion above of parameter (*t*) is assumed the thickness of low RI trenches change synchronously. So does the thickness of high RI rings (*d*) and the gap width (*t*_*gap*_). Next, we discuss the cases when there is only one parameter changing. Assume the fiber parameters are: *a* = 25 μm, *t* = 6 μm, *d* = 10 μm, *t*_*gap*_ = 18 μm, *Δn* = 0.007 and *R* = 15 cm. Fig 15A–15C show the losses of LP_01_, LP_11v_ and LP_11h_ modes and the mode of FM with different low RI trench thickness *t*_*1*_, *t*_*2*_ and *t*_*3*_, respectively. It can be seen that, *t*_*1*_, *t*_*2*_ and *t*_*3*_ has a small influence on the losses of LP_01_, LP_11v_ and LP_11h_ modes. *t*_*1*_ has a big influence on *A*_*eff*_ of FM. However, *t*_*2*_ and *t*_*3*_ has a small influence on *A*_*eff*_. The effect of one low RI trench is weak.

Fig 16(A) abd 16(B) show the losses of LP_01_, LP_11v_ and LP_11h_ modes and the mode are of FM with different high RI ring thickness *d*_*1*_ and *d*_*2*_, respectively. *d*_*1*_ and *d*_*2*_ have small influence on the losses of LP_01_, LP_11v_ and LP_11h_ modes and *A*_*eff*_ of FM. The effect of one high RI ring thickness is weak.

Fig 17A–17C show the losses of LP_01_, LP_11v_ and LP_11h_ modes and the mode are of FM with different gap width *t*_*g1*_, *t*_*g2*_ and *t*_*g3*_, respectively. *t*_*g1*_, *t*_*g2*_ and *t*_*g3*_ have small influence on the losses of LP_01_, LP_11v_ and LP_11h_ modes and *A*_*eff*_ of FM. *t*_*g1*_ has a big influence on *A*_*eff*_ of FM. However, *t*_*g2*_ and *t*_*g3*_ have small influence on *A*_*eff*_. The effect of one gap width is weak.

Numerical analysis results demonstrate that when there is only one parameter changing, the fiber confirms to single mode operation.

## Conclusion

A novel design of multi-trench fiber (MTF) with gaps is proposed and investigated in this paper. This fiber shows more excellent single-mode (SM) operation than standard MTF. The fiber can achieve mode area of 840 μm^2^ with high loss ratio (>300) under a tight bending radius of 15 cm. It has a special character that leakage loss decreases with the decreases of bending radius. The fiber can achieve better SM operation with smaller bending radius. The fiber performance is resistant to one parameter variation that, it allows small errors during practical fabrication. This design shows the potential of mode field scaling and makes a contribution to compact high power fiber lasers.

Our present work is based on theoretical analysis. We are currently fabricating this fiber and the actual performance will be tested in the near future.
